# Supplementary material for: Causal Relationship between Aging and Anorexia Nervosa: A White-Matter-Microstructure-Mediated Mendelian Randomization Analysis
Source: Biomedicines. 2024 Aug 16;12(8):1874. doi: 10.3390/biomedicines12081874 (PMC11351342; doi:10.3390/biomedicines12081874)
Supplement: Supplementary file 1 [file biomedicines-12-01874-s001.zip › Supplementary Figure/Figure S4/Singe SNP plot of MR analysis for TL to ubm-b-1692.pdf]

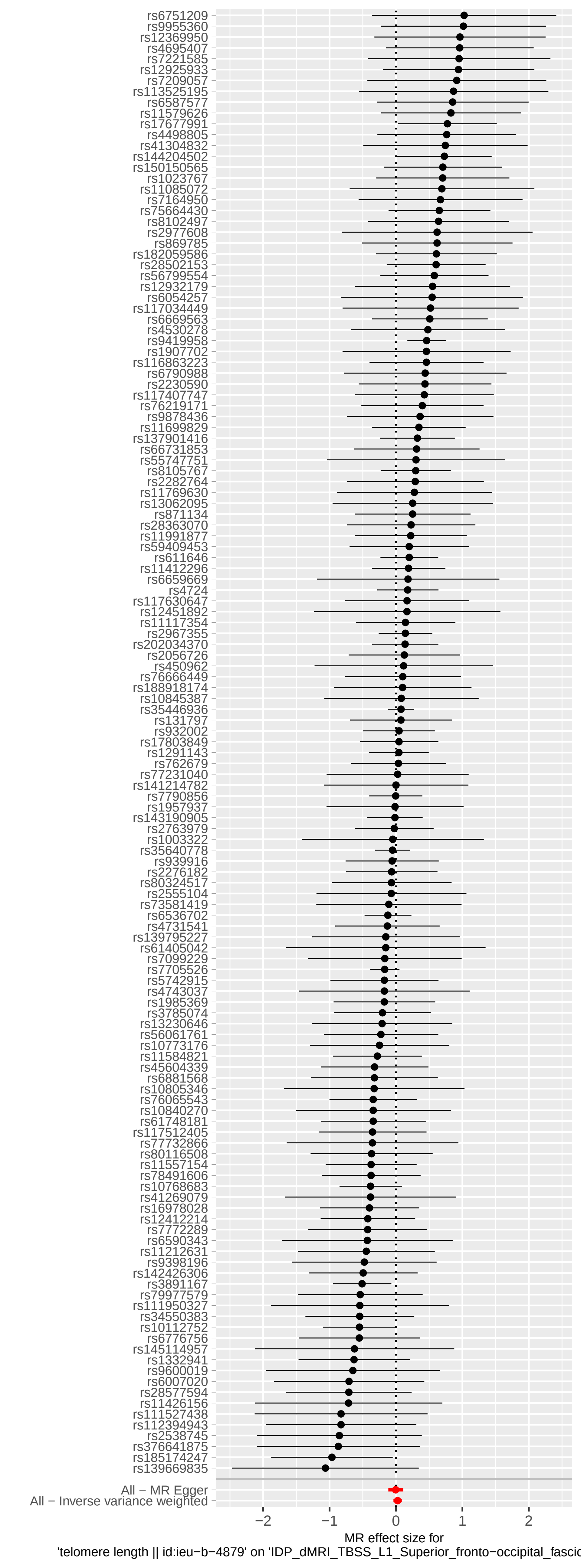
rs6751209
rs9955360
rs12369950
rs4695407
rs7221585
rs12925933
rs7209057
rs113525195
rs6587577
rs11579626
rs17677991
rs4498805
rs41304832
rs144204502
rs150150565
rs1023767
rs11085072
rs7164950
rs75664430
rs8102497
rs2977608
rs869785
rs182059586
rs28502153
rs56799554
rs12932179
rs6054257
rs117034449
rs6669563
rs4530278
rs9419958
rs1907702
rs116863223
rs6790988
rs2230590
rs117407747
rs76219171
rs9878436
rs11699829
rs137901416
rs66731853
rs55747751
rs8105767
rs2282764
rs11769630
rs13062095
rs871134
rs28363070
rs11991877
rs59409453
rs611646
rs11412296
rs6659669
rs4724
rs117630647
rs12451892
rs11117354
rs2967355
rs202034370
rs2056726
rs450962
rs76666449
rs188918174
rs10845387
rs35446936
rs131797
rs932002
rs17803849
rs1291143
rs762679
rs77231040
rs141214782
rs7790856
rs1957937
rs143190905
rs2763979
rs1003322
rs35640778
rs939916
rs2276182
rs80324517
rs2555104
rs73581419
rs6536702
rs4731541
rs139795227
rs61405042
rs7099229
rs7705526
rs5742915
rs4743037
rs1985369
rs3785074
rs13230646
rs56061761
rs10773176
rs11584821
rs45604339
rs6881568
rs10805346
rs76065543
rs10840270
rs61748181
rs117512405
rs77732866
rs80116508
rs11557154
rs78491606
rs10768683
rs41269079
rs16978028
rs12412214
rs7772289
rs6590343
rs11212631
rs9398196
rs142426306
rs3891167
rs79977579
rs111950327
rs34550383
rs10112752
rs6776756
rs145114957
rs1332941
rs9600019
rs6007020
rs28577594
rs11426156
rs111527438
rs112394943
rs2538745
rs376641875
rs185174247
rs139669835
All − MR Egger
All − Inverse variance weighted
−2
−1
0
1
2
MR effect size for
'telomere length || id:ieu−b−4879' on 'IDP_dMRI_TBSS_L1_Superior_fronto−occipital_fascic
